# Intravenous Thrombolysis in Chinese Patients with Different Subtype of Mild Stroke: Thrombolysis in Patients with Mild Stroke

**DOI:** 10.1038/s41598-017-02579-2

**Published:** 2017-05-23

**Authors:** Weiqi Chen, Yuesong Pan, Xingquan Zhao, Liping Liu, Hao Li, Xiaoling Liao, Chunjuan Wang, Yilong Wang, Yongjun Wang

**Affiliations:** 1 Department of Neurology, Beijing Tiantan Hospital, Capital Medical University, Beijing, China; 2 China National Clinical Research Center for Neurological Diseases (NCRC-ND), Beijing, China; 3 Center of Stroke, Beijing Institute for Brain Disorders, Beijing, China; 4 Beijing Key Laboratory of Translational Medicine for Cerebrovascular Disease, Beijing, China; 5 Department of Epidemiology and Health Statistics, School of Public Health, Capital Medical University, Beijing, China

## Abstract

Thrombolysis treatment for patients with mild stroke is controversial. The aim of our study was to investigate whether patients with mild stroke or its specific etiologic subtype might benefit from rt-PA therapy. Data were derived from two cohorts of patients with and without rt-PA treatment: (1) the Thrombolysis Implementation and Monitor of Acute Ischemic Stroke in China (TIMS-China) and (2) the China National Stroke Registry (CNSR) database. Patients with mild stroke (defined as National Institutes of Health Stroke Scale ≤5) receiving the rt-PA therapy and without rt-PA therapy were matched in 1:2 for age, sex, stroke severity and etiologic subtype. A total of 134 rt-PA-treated patients were matched to 249 non-rt-PA-treated patients in the study. Among them, 104 **(**76%) rt-PA-treated patients with mild stroke had good outcome after 3 months compared with 173 (69.5%) non-rt-PA-treated matching cases (odds ratio [OR], 1.48; 95% confidence interval [CI], 0.91–2.43; P = 0.12). Compared with non-rt-PA-treated group, rt-PA-treated patients had good outcome after 3 months in those with stroke subtype of large-artery atherosclerosis (LAA) (80.5% vs 65.1%; OR, 2.19; 95%CI, 1.14–4.21; P = 0.02). For patients with mild stroke, intravenous rt-PA treatment may be effective. Patients with stroke subtype of LAA might benefit more from rt-PA treatment.

## Introduction

Approximately 3 million new strokes occur every year in China, and 30% of them are mild ischemic strokes^1, 2^. About 10 to 20% of patients with mild stroke develop a new stroke within 3 months, and most of these recurrent strokes appear within 2 days after symptom onset^3–6^. The intravenous rt-PA treatment was considered as one of the most effective treatments for patients with acute ischemic stroke^7^. However, few patients with mild stroke receive intravenous rt-PA therapy^8^ because that thrombolysis treatment in acute phase to those patients is controversial^9^. Some physicians considered that the application of rt-PA in patients with mild symptoms could increase the risk of cerebral hemorrhage^10^. However, previous studies showed that 29% of patients with mild or rapidly improving symptoms not receiving thrombolysis led to poor outcome^11–13^. Up to now, only very few patients with mild stroke were included in randomized trials of rt-PA^14, 15^. Therefore, recurrence of stroke and other vascular events in patients with an initial mild stroke or TIA have become a frustrating medical situation^5, 16^. Furthermore, previous study reported that different stroke etiology subtypes appeared as independent risk predictors for early worsening in patients with mild ischemic stroke^17^. Little is known about the influence of stroke etiology on the effect of rt-PA treatment for patients with mild stroke^18^.

We investigated whether patients with mild ischemic stroke could benefit from intravenous rt-PA therapy by comparing matched pairs of patients with and without receiving rt-PA. We also hypnotized that the intravenous thrombolysis may have differential contribution to prognosis of the different subtypes of mild stroke.

## Results

### Patient Characteristics

**Figure 1:**
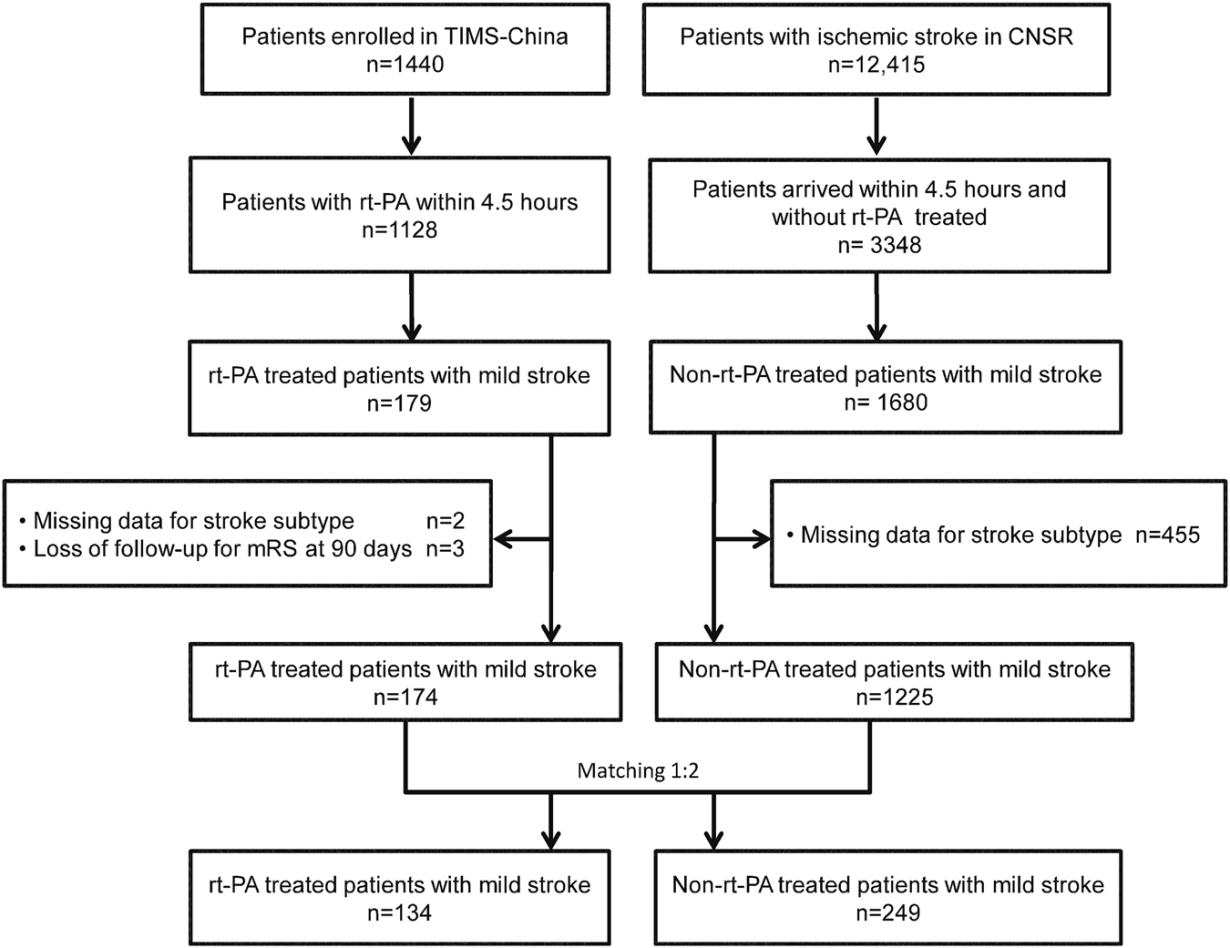
The flowchart of the TIMS-China and the CNSR cohorts. TIMS-China: Thrombolysis Implementation and Monitor of Acute Ischemic Stroke in China; CNSR: China National Stroke Registry.

A total of 174 rt-PA treated patients with mild stroke and with complete baseline variables were identified in the Thrombolysis Implementation and Monitor of Acute Ischemic Stroke in China^19^ TIMS-China) registry, while 1225 non-rt-PA treated patients with mild stroke in the China National Stroke Registry (CNSR) registry^20^. According to the predefined matching method, we matched 134 rt-PA treated patients to 249 non-rt-PA treated patients in our study (Fig. 1).

The mean age of rt-PA treated patients was 62.3 ± 10.5, and was 63.2 ± 10.6 in rt-PA untreated patients (Table 1). There were 48 patients (35.8%) in rt-PA treated group and 90 patients (36.1%) in rt-PA untreated group were female. A total of 68.7% of the patients had a history of hypertension, 17.9% had diabetes mellitus, and 7.5% had atrial fibrillation in rt-PA treated group, and the proportions were 62.7%, 23.7% and 8.4% in untreated group respectively. However, patients in untreated group had a higher proportion of history of stroke (P < 0.001).

**Table 1:** Baseline Characteristics of Matched Patients with Mild Stroke.

Characteristics	rt-PA(n = 134)	Non-rt-PA(n = 249)	p
Female, n (%)	48(35.8)	90(36.1)	1.00
Age, y (mean ± SD)	62.3 ± 10.5	63.2 ± 10.6	0.06
Hypertension, n (%)	92(68.7)	156(62.7)	0.23
Diabetes Mellitus, n (%)	24(17.9)	59(23.7)	0.20
Atrial Fibrillation, n (%)	10(7.5)	21(8.4)	0.25
History of stroke, n (%)	23(17.2)	89(35.7)	<0.001
Smoking, n (%)	63(47.0)	71(28.5)	<0.001
Pre- stroke mRS 0–1	129(96.3)	234(94.7)	0.51
NIHSS, median (IQR)	4(4–5)	4(4–5)	1.00
Stroke subtype, n (%)			0.82
Large artery atherosclerosis	77(57.5)	152(61.0)	
Small vessel occlusion	30(22.4)	56(22.5)	
Cardioembolism	11(8.2)	18(7.2)	
Other/Undetermined	16(11.9)	23(9.2)	
Treatment during hospitalization, n (%)			
Anti-platelet	121(90.3)	229(92.0)	0.86
Anticoagulants	29(21.6)	77(30.9)	0.01
Antihypertensive	57(42.5)	129(51.8)	0.06

IQR: interquartile range; mRS: modified Rankin Scale; NIHSS: National Institutes of Health Stroke Scale; rt-PA: recombinant tissue plasminogen activator; SD: Standard Deviation.

### Outcome

We compared the outcomes at 3 months after mild stroke onset in rt-PA treated patients with matched rt-PA untreated patients.

**Figure 2:**
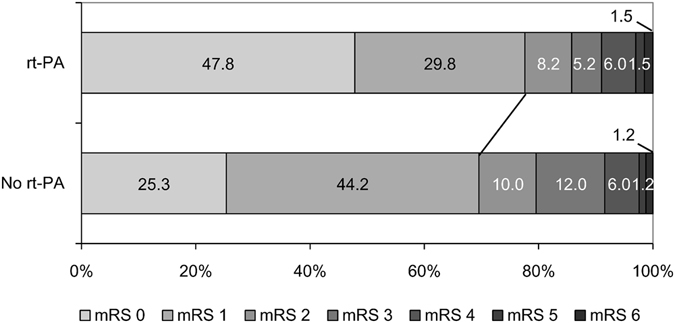
Distribution of mRS scores at 3 months in matched patients with mild stroke with and without rt-PA treatment. mRS: modified Rankin Scale; rt-PA: recombinant tissue plasminogen activator.

Figure 2 showed the distribution of mRS score at 3 months in matched patients with mild stroke. We found a shift toward upgraded outcome in rt-PA treated patients with mild stroke compared with rt-PA untreated patients. There were 77.6% of the patients in rt-PA treated group having good functional outcome (mRS 0–1) at 3 months, while the proportion in the untreated group was 69.5%. We found a favorable trend of better outcome in rt-PA treated patients, although the overall difference was not statistically significant (OR, 1.48; 95% CI, 0.91–2.43; P = 0.12).

**Figure 3:**
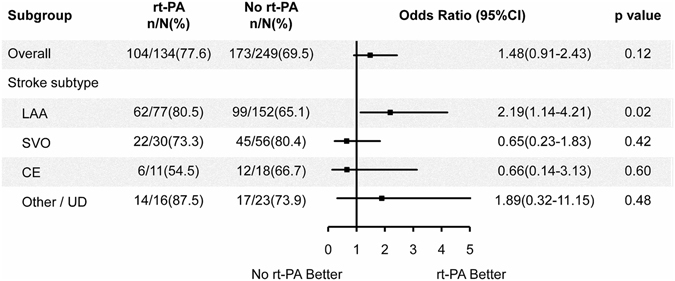
Odds ratio of rt-PA treatment for patients with mild stroke. CE: cardioembolism; CI: confidence interval; LAA: large artery atherosclerosis; mRS: modified Rankin Scale; rt-PA: recombinant tissue plasminogen activator; SVO: small vessel occlusion; UD: undetermined.

In the subgroup analysis according to stroke subtype (Fig. 3), 80.5% (62/77) rt-PA treated patients with LAA had good outcome (mRS 0–1) at 3 months, compared with 65.1% (99/152) rt-PA untreated patients with LAA had good outcome at 3 months after stroke onset (OR, 2.19; 95% CI, 1.14–4.21; p = 0.02). This benefit effect was not observed in the SVO, CE and “other” determined etiology and UD subtype groups (p = 0.42, p = 0.60 and p = 0.48, respectively). The ORs with 95% CIs of rt-PA treated and untreated groups are shown in Fig. 3.

The mortality rate was 1.5% (2 of 134) in rt-PA-treated patients, while 1.2% (3 of 249) in patients without rt-PA treatment (OR, 1.00; 95% CI, 0.16–6.14; p = 1.00). Only one (0.7%) patient in rt-PA treatment group had symptomatic intracranial hemorrhage (sICH) defined by ECASS II (second European- Australasian acute stroke study) criteria during three months.

## Discussion

This observational study showed that mild stroke patients who received intravenous rt-PA treatment obtained a high rate (77.6%) of good outcome (mRS 0–1 at 3 months), which was similar with previous study reported^21^. We also found that patients with mild stroke whose etiological type was LAA might benefit more from the intravenous rt-PA than other types of etiology.

The sICH incidence in mild stroke patients receiving rt-PA treatment in other studies have been reported in a range of 0% to 3.7%^22, 23^. Considering the baseline NIHSS was closely associated with the incidence of sICH in patients treating with rt-PA^24^, the sICH rate was considered to be low in mild stroke patients. In the present study, only one patient had sICH (0.7%) in rt-PA treatment group. Meanwhile, the sICH rate of patients despite of the baseline NIHSS in the overall TIMS-China study was 3.1%^25^. Compared with the study conducted in Korea^26^ with 4.1% of SICH incidence in rt-PA group, the SICH rate in our study was very low. The reason might be related to the small sample size of our study.

This analysis allowed us to estimate the effect of rt-PA in the mild stroke patients who were excluded from most randomized trials. Previous results from randomized studies^14, 15^ and observational cohorts^27–29^ demonstrated controversial results about the effectiveness of intravenous rt-PA treatment in patients with mild stroke. Our study provided data that patients with mild stroke might potentially benefit from intravenous thrombolysis, especially in those with LAA etiology subtype. It is still unknown which criteria should be used for choosing the appropriate candidates for intravenous rt-PA treatment among patients with mild stroke^26^. We knew that patients with LAA were more likely to experience symptom worsening than other types of etiology^30^. Although, in clinical practice, the etiology of the stroke usually could not be accurately determined within the short “time-window” of rt-PA treatment, we found that mild stroke patients with LAA might benefit more from rt-PA treatment than standard care.

Also, well design randomized clinical trials or larger observational studies are needed to confirm the effectiveness of intravenous thrombolytic therapy in mild ischemic stroke patients. However, given the large sample sizes required, this will create a major challenge for the design and recruitment of future randomized controlled trials. The Potential for rt-PA to Improve Strokes with Mild Symptoms (PRISMS, NCT02072226) trial^31^ which has closed, was designed as a double-blinded, randomized trial evaluating the efficacy and safety of intravenous rt-PA vs. aspirin 325 mg in mild stroke patients (NIHSS ≤ 5). The results of this trial are expected in the near future.

Our study also had several limitations. First, only 14.8% of patients in the CNSR registry were included in this analysis after paired matching, which might indicate potential selection bias of our study. Second, considering the fact that we only included 300 subjects in this study, it had limited power to detect the effects of thrombolysis among patients with mild stroke. The small sample size was also the potential explanation of the statistically insignificant results of the present study. Third, our study was an observational study and post hoc analysis was performed for stroke subtype; therefore, it could not provide adequate level of evidence of the overall benefit of intravenous rt-PA in mild stroke patients. The results should be further validated in well-designed randomized clinical trial. Fourth, we only reported effectiveness of rt-PA treatment in mild stroke patients, without safety index (sICH) in the rt-PA untreated patients. Finally, the matching process did not control for the variables such as the nature of the hospital or the nature of the treating physician, and the rt-PA untreated group had a higher history of stroke. This might cause bias and undermined the conclusion.

In conclusion, administering intravenous rt-PA to patients with mild stroke (NIHSS ≤ 5) may lead to potential better clinical outcome compared to not receiving thrombolysis treatment. Patients with stroke subtype of LAA might be likely to benefit from intravenous thrombolysis therapy than other stroke subtypes but need further study to verify it.

## Methods

Patients receiving rt-PA treatment were derived from the TIMS-China^19^. Patients not receiving rt-PA treatment were derived from the CNSR^20^ database which was conducted as the same period as TIMS-China. From both cohorts, we extracted the following variables: age, sex, medical history (including hypertension, diabetes mellitus, atrial fibrillation, history of stroke, smoking), stroke severity (measured by National Institutes of Health Stroke Scale, NIHSS), stroke subtype and treatment during hospitalization, *et al*. The detailed information of screening process was showed in Fig. 1.

The good outcome of patients with mild stroke was defined as a modified Rankin Scale (mRS) of 0 to 1 at 3 months^21, 26, 32^. All patients with acute mild ischemic stroke were further classified according to the TOAST (Trial of Org 10172 in Acute Stroke Treatment)^33^ criteria: large-artery atherosclerosis (LAA), small-vessel occlusion (SAO), cardioembolism (CE), stroke of other determined etiology (other), and stroke of undetermined pathogenesis (UD)^33^. The overall inter-rater agreement for the TOAST classification was good (κ value of 0.73 [95% CI, 0.65–0.81])^34^.

### TIMS-China Cohort

TIMS-China was a national prospective stroke registry of thrombolytic therapy with intravenous rt-PA (Actilyse, Boehringer Ingelheim, Germany) in patients with acute ischemic stroke in China^19^. The registry enrolled 1440 consecutive patients with rt-PA treatment from 67 centers in China since May 2007 to April 2012. Data on clinical characteristics, computer tomographic (CT) or magnetic resonance imaging (MRI) scans of brain, medical therapy and intravenous thrombolysis information were collected. The data in the TIMS-China was gathered by experienced physicians with standard case report form after obtained informed consent for participating in the registry and receiving thrombolysis therapy. The follow up duration was 3 months through face to face or telephone.

### CNSR Cohort

Between September 2007 and August 2008, 22 216 adult patients from 132 participating hospitals with acute stroke were recruited into the CNSR which was the first large scale nationwide stroke registry in China^20^. Detailed baseline data were collected through using paper-based case report forms. Trained stroke neurologists or research personnel performed data collection at baseline, 3 months, 6 months and 12 months. The interviewers were not allowed to work until having passed examination and obtained certification for the NIHSS and modified Rankin Scale (mRS). Written informed consent was obtained from all patients or their legal representatives.

### Matching

Patients with mild stroke with intravenous rt-PA and without intravenous rt-PA treatment were 1:2 matched according to age, gender, stroke severity at baseline (according to NIHSS), stroke subtype (according to the TOAST criteria). We permitted a ±5 years for age between the two groups. We enforced exact matching for the nominal level of measurement for gender and stroke subtype (TOAST criteria). For stroke severity, we enforced exact matching from 0 to 5 points of the NIHSS. Two patients in the control group was extracted by random sampling technique if more than 3 cases were matched.

### Statistical Analysis

Continuous variables were compared with the Student’s t test or Mann-Whitney tests as appropriate. Categorical variables were compared with Pearson’s χ^2^ or Fisher’s exact tests. Odds ratios (ORs) with their confidence intervals (CIs) for good outcome were estimated using conditional logistic regression models in total patients and by stroke subtype, respectively. The level of significance was established at a two-tailed value of p < 0.05. All analyses were processed with SAS software version 9.3 (SAS Institute Inc, Cary, NC).

### Ethical approval

The study was approved by both the Ethics Committee of the Beijing Tiantan Hospital, in compliance with the Declaration of Helsinki. Written informed consent was obtained from all participants. All the experiments described were performed in accordance with the approved guidelines.
